# Transactions of the American Roentgen Ray Society

**Published:** 1903-09

**Authors:** 


TRANSACTIONS OF THE AMERICAN ROENTGEN RAY SOCIETY. Third annual meeting held at Chicago, Ill., December 10 and 11, 1902. James B. Bullitt, M. D., Secretary. (Price: cloth, $1.25; paper, 75 cents.)

This society was organised for the purpose of putting on record the most striking successes of the new light science. The book contains 14 papers, all upon the subjects connected with radiotherapy. The use of the *x*-ray and its allies is becoming more and more common; so common, indeed, as to preclude the necessity of a national organisation on the part of those employing these agents. Used as it is in all departments of medical science it would not seem to demand any such distinction. The medical profession has already been nationally organised to suffocation. Let us not seek to add anything to the present alarming aggregate. G. W. W.

